# Unintended pregnancy and associated factors among pregnant women in Arsi Negele Woreda, West Arsi Zone, Ethiopia

**DOI:** 10.1186/s13104-018-3778-7

**Published:** 2018-09-17

**Authors:** Robera Olana Fite, Abdurahman Mohammedamin, Tilaye Workneh Abebe

**Affiliations:** 1 Department of Nursing, College of Medicine and Health Sciences, Wolaita Sodo University, PO-Box: 138, Wolaita Sodo, Ethiopia; 2 Department of Public Health, College of Health Sciences, Adama General Hospital and Medical College, Adama, Ethiopia

**Keywords:** Unintended pregnancy, Arsi Negele, Unplanned

## Abstract

**Objective:**

The study was aimed at determining the prevalence of unintended pregnancy and associated factors in Arsi Negele Woreda from May 01, 2017 to July 30, 2017.

**Results:**

Unintended pregnancy was found to be 41.5%. The multivariable logistic regression revealed that 35 and above age group (AOR; 2.343, 95% CI 1.374, 3.997), single marital status (AOR; 6.492, 95% CI 1.299, 32.455), parity of 2 (AOR; 53.419, 95% CI 21.453, 133.014), parity of 3 and above (AOR; 20.219, 95% CI 7.915, 51.655), having abortion history (AOR; 1.962, 95% CI 1.025, 3.755), having health professional visit (AOR; 2.004, 95% CI 1.218, 3.298) and having autonomy to use contraceptive method (AOR; 2.925, 95% CI 1.648, 5.190) were significantly associated with unintended pregnancy. Therefore, reproductive health advocacy, counseling and access of modern contraceptive methods are recommended.

**Electronic supplementary material:**

The online version of this article (10.1186/s13104-018-3778-7) contains supplementary material, which is available to authorized users.

## Introduction

World Health Organization (WHO) defined reproductive health as a state of complete physical, mental and social well-being, and not merely the absence of disease in all matters relating to the reproductive system and its function. It encompasses the freedom to control reproduction [1]. Uncontrolled sexual activity may result in unintended pregnancy. An Unintended pregnancy is a major health concern. It has association with higher mortality and morbidity of both mothers and newborns. The level of unintended pregnancy implies women’s reproductive health status [2–4]. An unintended pregnancy is unwanted or mistimed pregnancy. A mistimed pregnancy occurs in women who conceived sooner or later than the desired time. An unwanted pregnancy happens in women who do not want any more children at the time of conception [5, 6]. In Ethiopia, 17% and 8% pregnancies were mistimed and unwanted, respectively [7].

According to the Ethiopian Health sector transformation plan report, maternal and newborn health is a major priority. Reducing the number of unintended pregnancies promotes reproductive health [7, 8].

Unintended pregnancies affect children’s health. Hence, prevention is important to reduce maternal and infant mortality [9]. Accordingly, the national reproductive health program plans to reduce the maternal death to 199 per 100,000 live births and neonatal mortality rate to 10 per 1000 live births by 2010 [7].

Various barriers expose women to unintended pregnancy. The barriers are related to the health institutions accessibility, autonomy, reproductive health-related knowledge and health service quality [4].

Health sectors have tried to address the issue of unintended pregnancy. However, it is still a major health-related challenge. In sub-Saharan African countries, unintended pregnancy rate is high [8, 10, 11]. In developing countries, 49% of the 21 million pregnancies of women aged 15–19 is unintended pregnancy [12].

Unintended pregnancy mainly results from inconsistent use or not using contraceptive methods [13]. Although family planning demand among reproductive age group women has increased over time, the use is not satisfactory [13, 14]. In Ethiopia, unmet need for contraceptive methods is 22% [7].

Women might have an abortion due to unintended pregnancy [5, 9]. The level of abortion in a country implies the magnitude of unintended pregnancy. In Ethiopia, the annual abortion rate is 28 per 1000 reproductive age group women [15, 16]. Globally, 86 million pregnancies were unintended, of which 41 million resulted in abortions [11].

Addressing factors contributing to unintended pregnancy is essential to ensure safe and reliable service to reproductive age group women. Finding from this study will be helpful for health professionals, policymakers, and stakeholders in understanding factors associated with unintended pregnancy and designing possible interventions.

## Main text

### Methods

#### Study area, period and design

The study was conducted from in Arsi Negele Woreda. Arsi Negele Woreda is found in Oromia regional state. It is 220 km far from the capital city of Ethiopia, Addis Ababa. It has a total population of 77,942 of which 49.49% are males and 50.5% are females. The Woreda has 1 private hospital, 2 health centers and 5 private clinics. A community based cross sectional study design was employed from May 01–July 30, 2017.

#### Population and eligibility criteria

The source of population was all pregnant women living in Arsi Negele Woreda. The study sample was selected pregnant women living in Arsi Negele Woreda and the study units were individuals at household levels.

Pregnant women residing for at least 6 months were included in the study. Those who are critically ill and mentally challenged were excluded from the study.

#### Sample size and sampling technique

The sample size was determined using a formula for estimation of single population proportion with the following assumptions: 36.5% proportion [17], Z *a/2* is the Z value at 95% Confidence level (1.96) and 0.05 margin of error (d). Since the source population is ˂ 10,000 finite population correction formula was used. Adding 10% for the non-response rate and considering design effect of two the final sample size was 704. The study employed multi-stage sampling technique. Four villages were selected by lottery method from eight villages. After getting the sampling frame in each village, the final sample size was proportionally allocated based on their respective population. Finally the study units were selected by systematic random sampling technique until the desired sample size for each village attained.

#### Operational definitions

##### Unintended pregnancy

Is unwanted pregnancy or mistimed pregnancy [17, 23, 28, 29]. It was assessed through a question asking women whether their pregnancy is entirely unwanted or wanted but at a later time. Response included the two categories: (1) No, (2) Yes. Therefore, if the women reported that she didn’t want the pregnancy or wanted pregnancy but at a later time, the pregnancy was considered as unintended.

##### Health professional visit

It was assessed through a question asking reproductive health professional contact before pregnancy.

##### Contraceptive utilization

It was assessed through a question asking the use of any contraceptive method before pregnancy.

##### Autonomy to choose contraceptive method

Refers to the power to give decision on selecting contraceptive method.

#### Data collection tool and procedure

The structured questionnaire is adapted after a review of different literature [17–19]. The questionnaire sought information on respondents’ socio-demographic characteristics, obstetrics history and contraceptive utilization history. Four data collectors who are a fluent speaker of the Afan Oromo language were recruited as a data collector. Training was provided for 2 days by the principal investigator.

#### Data quality assurance

The data collection tool was translated to Afan Oromo and Amharic language. After that, it was retranslated to the English version to check its consistency. Pretesting of the data collection tools was conducted. Following the pretest, the tool was improved. Adequate training and supervision was provided.

#### Data analysis procedures

Data first was checked manually for completeness and then each completed questionnaire was assigned a unique code. Subsequently the data was entered into Epidata version 3.1. The generated data was transferred to statistical Package for Social Sciences (SPSS) version 20. All explanatory variables that have association in bivariate analysis with p-value less than 0.25 was entered into multivariable logistic regression model. p-value less than 0.05 was taken as significant association.

### Results

#### Socio-demographic characteristics

A total of 644 pregnant women participated in the study, obtaining a response rate of 91.5%. More than three-fourth of the respondents (80.1%) were aged 17–34. More than half (95.2%) were married and 523 (81.2%) attended primary education. Majority of the respondent’s husband (87.3%) attended primary education. Regarding Occupation, 337 (52.3%) were housewives. The difference in the level of unintended pregnancy by age group was statistically significant (Table 1 and Additional file 1: Figure S1).

**Table 1:** Socio-demographic characteristics of pregnant women in Arsi Negele Woreda, West Arsi Zone, Ethiopia, 2017

Variable	Frequency	Percent	Unintended pregnancy	p-value
			N (%)	
Age				
17–34	516	80.1	31 (6)	< 0.001
35 and above	128	19.9	97 (75.8)	
Age at first pregnancy				
Less than 18	81	12.6	32 (39.5)	0.703
18 and above	563	87.4	235 (41.7)	
Marital status				
Married	613	95.2	246 (40.1)	0.005
Single	14	2.2	11 (78.6)	
Divorced/widowed	17	2.6	10 (58.8)	
Women educational status				
No formal education	100	15.5	47 (47)	0.962
Primary	523	81.2	206 (39.4)	
Secondary and above	21	3.3	14 (66.7)	
Husband educational status				
No formal education	54	8.4	19 (35.2)	0.688
Primary education	562	87.3	238 (42.3)	
Secondary and above	28	4.3	10 (35.7)	
Religion				
Orthodox	131	20.3	54 (41.2)	0.884
Muslim	361	56.1	150 (41.6)	
Protestant	108	16.8	47 (43.5)	
Catholic	33	5.1	11 (33.3)	
Others	11	1.7	5 (45.5)	
Ethnicity				
Oromo	260	40.4	113 (43.5)	0.402
Amhara	243	37.7	90 (37)	
Tigire	78	12.1	39 (50)	
Keffa	45	7	17 (37.8)	
Woliata	14	2.2	6 (42.9)	
Others	4	0.6	2 (50)	

#### Obstetrics history

Of the respondents, 267 (41.5%) and 377 (58.5%) had unintended and intended pregnancy respectively. Three hundred twenty-five (50.5%) and 334 (51.9%) had gravidity and parity of 2, respectively. Two hundred ninety-seven (46.1%) had birth interval of 1–2 years. Only 41 (15.4%) reported lack of knowledge as a reason for unintended pregnancy. Majority (86.5%) had abortion history and 73 (11.3%) had one abortion. Seventy-nine (12.3%) got abortion care service from government facility and 393 (61%) had a discussion with their husband about the contraceptive methods. Most of them (71%) had autonomy to use contraceptive method and 456 (70.8%) had health professional visit. More than half (66.5%) ever used contraceptives and 122 (18.9%) used contraceptives in the last 12 months. Eighteen (2.8%), 77 (12%), 7 (1.1%), 18 (2.8%), 2 (0.3%) used Pills, Injectable, condoms, Implants, IUCD, respectively (Table 2).

**Table 2:** Obstetrics history of pregnant women in Arsi Negele Woreda, West Arsi Zone, Ethiopia, 2017

Variable	Frequency	Percent
Pregnancy intention		
Unintended	267	41.5
Intended	377	58.5
Gravidity		
1	196	30.4
2	325	50.5
3 and above	123	19.1
Parity		
1	190	29.5
2	334	51.9
3 and above	120	18.6
Preceding birth interval		
Less than 1 year	262	40.9
1–2 years	297	46.1
Greater than 2 years	85	13
Reason of unintended pregnancy (n = 267)		
Lack of knowledge	41	15.4
Side effect of fears	53	19.9
Contraceptive failure	65	24.3
Can’t get contraceptive	3	1.1
Breast feeding	54	20.2
Not allowed to use contraceptive	20	7.5
Health related problem	12	4.5
Have no reason	39	14.6
Abortion history		
Yes	87	13.5
No	557	86.5
Abortion number (n = 87)		
1	73	11.3
2	11	1.7
More than 2	3	0.5
Abortion place (n = 87)		
Government facility	79	12.3
Private facility	4	0.6
NGO	1	0.2
Traditional place	3	0.5
Discussion about contraceptive with husband		
Yes	251	39.0
No	393	61.0
Autonomy to use contraceptive method		
Yes	457	71
No	187	29
Health professionals visit		
Yes	456	70.8
No	188	29.2
Ever used contraceptive		
Yes	428	66.5
No	216	33.5
Used contraceptive in last 12 months		
Yes	122	18.9
No	522	81.1
Contraceptive method used (n = 122)		
Pills	18	2.8
Injectable	77	12
Condom	7	1.1
Implant	18	2.8
IUCD	2	0.3

#### Factors associated to the unintended pregnancy

Age, marital status, parity, abortion history, health professional visit and autonomy to use contraceptive method were found to be significant predictors of unintended pregnancy. Accordingly, pregnant women in the age group 35 and above were 2 times more likely to experience unintended pregnancy than pregnant women in the age group of 17–34 (AOR; 12.343, 95% CI 1.374, 3.997). Single women were 6 times less likely to experience unintended pregnancy than pregnant women who were married (AOR; 6.492, 95% CI 1.299, 32.455). Pregnant women with a parity 2 were 53 times more likely to experience unintended pregnancy than pregnant women with a parity of 1 (AOR; 53.419, 95% CI 21.453, 133.014). In addition, Pregnant women with a parity of 3 and above were 20 times more likely to experience unintended pregnancy than pregnant women with a parity of 1 (AOR; 20.219, 95% CI 7.915, 51.655). Pregnant women who had no abortion history were 2 times more likely to experience unintended pregnancy than pregnant women who had abortion history (AOR; 1.962, 95% CI 1.025, 3.755). Pregnant women who had no health professional visit were 2 times more likely to experience unintended pregnancy than pregnant women who had health professional visit (AOR; 2.004, 95% CI 1.218, 3.298). Pregnant women who had no autonomy to use contraceptive method were 3 times more likely to experience unintended pregnancy than pregnant women who had autonomy (AOR; 2.925, 95% CI 1.648, 5.190) (Table 3).

**Table 3:** Factors associated to unintended pregnancy of pregnant women in Arsi Negele Woreda, West Arsi Zone, Ethiopia, 2017

Variables	Unintended pregnancy		COR (95% CI)	AOR (95% CI)
	No	Yes		
Age				
17–34	346	170	1	1
35 and above	31	97	6.369 (4.085, 9.929)*	2.343 (1.374, 3.997)**
Marital status				
Married	367	246	1	1
Single	3	11	5.470 (1.511, 19.808)**	6.492 (1.299, 32.455)**
Divorced/widowed	7	10	2.131 (0.800, 5.675)	0.728 (0.237, 2.233)
Age at first pregnancy				
Less than 18	49	32	1	1
18 and above	328	235	1.097 (0.682, 1.766)	0.830 (0.428, 1.609)
Parity				
1	181	9	1	1
2	117	217	37.300 (18.410, 75.573)*	53.419 (21.453, 133.014)*
3 and above	79	41	10.437 (4.841, 22.505)*	20.219 (7.915, 51.655)*
Abortion history				
Yes	59	28	1	1
No	318	239	1.584 (0.980, 2.559)	1.962 (1.025, 3.755)**
Health professional visit				
Yes	305	151	1	1
No	72	116	3.254 (2.287, 4.631)*	2.004 (1.218, 3.298)**
Contraceptive utilization				
Yes	215	213	1	1
No	162	54	0.336 (0.234, 0.483)*	1.107 (0.554, 2.215)
Autonomy to choose contraceptive method				
Yes	285	172	1	1
No	92	95	1.711 (1.214, 2.412)*	2.925 (1.648, 5.190)*

* Significant at p < 0.001; ** significant at p < 0.05

### Discussion

An unintended pregnancy is an important public health problem that predisposes women to maternal deaths and illnesses mainly through unsafe abortions and poor maternity care [20]. Therefore, identifying the level of unintended pregnancy is essential. The study assessed the unintended pregnancy and associated factors.

The prevalence of unintended pregnancy in the study was 41.5%. This is higher than the prevalence of unintended pregnancy in Hosana 34% [21], Welkaite 26% [22], Kenya 24% [23], Senegal 14.3% [24], Pakistan 38.2% [25] and 27% Canada [26]. This might be due to the differences in socio-cultural characteristics and health coverage. Difference in the availability and accessibility of maternal health services, including access to modern contraceptives among the study areas may contribute to the difference.

Pregnant women with parity of 3 and above were 20 times more likely to experience unintended pregnancy than pregnant women with a parity of 1. Similarly, studies from United States and Brazil have found that women who have more alive children are more likely to experience an unintended pregnancy [27, 28]. If the women have enough children, the intention for the next pregnancy will decrease. High parity woman might already have adequate children. In addition, it might imply the gaps in counseling and postpartum contraceptives provision.

Single women were 6 times less likely to experience unintended pregnancy than pregnant women who were married. This is supported by research from Ethiopia [29]. Single women engage in sexual activity for pleasure. Therefore, if pregnancy occurs it is more likely to be unintended. Furthermore, they are less likely to use contraceptive methods [30].

Advanced age has a positive association with unintended pregnancy. Pregnant women in the age group 35 and above were 2 times more likely to experience unintended pregnancy than pregnant women 17–34 years old. This result is supported by research from Pakistan and Canada [25, 31]. The effect of age on unintended pregnancy can be explained by the fact that as women grow older, they do not want any more children. But due to unmet need for contraceptives, these women experience unintended pregnancies.

The data also reveal that pregnant women who had no health professional visit were 2 times more likely to experience unintended pregnancy than pregnant women who had health professional visit. An unintended pregnancy and its negative consequences can be prevented through access to information from health professionals. Education regarding family planning service and supply points helped to decrease the level of unintended pregnancy. This is related to their role in providing information on family planning and increasing the utilization [32, 33].

### Conclusion

The study has shown that the level of unintended pregnancy is higher. Age, marital status, parity, abortion history, health professional visit and autonomy to use contraceptive method were significantly associated with unintended pregnancy. Strengthening reproductive health advocacy, counseling and increasing access of modern contraceptive method is essential to combat the problem. Furthermore, community health education to the reproductive age group women is important.

## Limitation

It has to be noted that the finding of this study mainly reflect situation in Arsi Negele Woreda Therefore, the findings should be interpreted with caution. The responses might have been liable to social desirability bias. The factors expected to influence unintended pregnancy may not be exhaustive.

## Additional file

**Additional file 1: Figure S1.** Occupation of pregnant women in Arsi Negele Woreda, West Arsi Zone, Ethiopia, 2017.

## Abbreviations

AOR: adjusted odds ration
CI: confidence interval
COR: crude odds ration
WHO: World Health Organization
