# Supplementary material for: Unintended pregnancy and associated factors among pregnant women in Arsi Negele Woreda, West Arsi Zone, Ethiopia
Source: BMC Res Notes. 2018 Sep 17;11:671. doi: 10.1186/s13104-018-3778-7 (PMC6142678; doi:10.1186/s13104-018-3778-7)
Supplement: Supplementary file 1 — Additional file 1: Figure S1. Occupation of pregnant women in Arsi Negele Woreda, West Arsi Zone, Ethiopia, 2017. [file 13104_2018_3778_MOESM1_ESM.docx]

Figure S1: Occupation of pregnant women in Arsi Negele Woreda, West Arsi Zone, Ethiopia, 2017
